# Olfaction and Aging: A Review of the Current State of Research and Future Directions

**DOI:** 10.1177/20416695211020331

**Published:** 2021-06-26

**Authors:** Jonas K. Olofsson, Ingrid Ekström, Maria Larsson, Steven Nordin

**Affiliations:** Gösta Ekman Laboratory, Stockholm University, Stockholm, Sweden; Department of Psychology, Stockholm University, Stockholm, Sweden; Aging Research Center, Karolinska Institutet and Stockholm University, Stockholm, Sweden; Gösta Ekman Laboratory, Stockholm University, Stockholm, Sweden; Department of Psychology, Stockholm University, Stockholm, Sweden; Department of Psychology, Umeå University, Umeå, Sweden

**Keywords:** chemosensory, development, lifespan/aging, memory, perception

## Abstract

Olfaction, the sense of smell, is characterized by a notable age-dependency such that aging individuals are more likely to have poor olfactory abilities. These impairments are considered to be mostly irreversible and as having potentially profound effects on quality of life and food behavior, as well as constituting warning signs of mortality, cognitive dysfunction, and dementia. Here, we review the current state of research on aging and olfaction, focusing on five topics which we regard to be of particular relevance for the field: nutrition and health, cognition and dementia, mortality, environment and genetics, and training-based enhancement. Under each of these headlines, we provide a state-of-the-art overview and discuss gaps in our knowledge which might be filled by further research. Understanding how olfactory abilities are diminished in aging, and how they may be alleviated or recovered, involves a set of challenging tasks for researchers in the years to come.

## Introduction

Smell loss is an invisible impairment in many aging persons. Whereas all sensory systems undergo changes with age, the changes observed in the olfactory system have unique causes as well as unique consequences for health and well-being. There is no corrective technology for olfaction that would correspond to hearing aids or eye-glasses. However, age-related olfactory changes are neither ubiquitous, nor are they always irreversible. There is reason to discuss these age-related changes in depth. In this review, we focus on some of the major topics in research on olfactory function in aging and provide recommendations for future research. We will initially provide a state-of-the-art review of age-associated olfactory changes, to be followed by an overview of five emerging topics which are deemed to be of particular interest, and for which new developments are to be expected in the coming years. These topics are nutrition and health, cognition and dementia, mortality, environment and genetics, and training-based enhancement. As our review spans a wide range of topics, it is not exhaustive, yet it discusses many of the key concepts, references, and emerging trends in the field.

Olfaction is multifaceted, and is often assessed by sensory detextion thresholds (differentiating faint odors from no-odor control stimuli), discriminating between odor qualities (detexting a deviant quality among identical control stimuli), and cued identification of everyday odors (accurately matching odors to labels in a list; Doty et al., 1984; Oleszkiewicz et al., 2019). Identification of everyday odors may also be assessed without the provision of contextual cues (i.e., free odor identification or odor naming) which is a cognitively effortful task (Larsson et al., 2016; Olofsson et al., 2013). A vast number of studies demonstrated impaired olfactory functions in aging (Choi et al., 2018; Hedner et al., 2010; Hummel et al., 1997; Kern et al., 2014; Larsson et al., 2000; Larsson, Hedner, et al., 2009; Schubert et al., 2017; Stuck et al., 2006). Age-related impairment is pronounced in odor quality discrimination (Choudhury et al., 2003; Hedner et al., 2010; Hummel et al., 1997; Kaneda et al., 2000) as well as in odor intensity ratings (S. Koskinen & Tuorila, 2005). Diminished odor intensity perception, as well as pronounced olfactory adaptation and slower recovery from adaptation, was suggested already in early work on aging and olfaction (J. C. Stevens & Cain, 1985, 1987; J. C. Stevens et al., 1982, 1989; Wysocki & Gilbert, 1989). Interestingly, psychophysical studies suggest largely independent, parallel processing of odor intensity and quality discrimination, as well as of odor detextion and quality discrimination (Cain et al., 2008; Rawson et al., 1998; Savic et al., 2000; de Wijk & Cain, 1994). Hence, for example, a mild-to-moderate loss in detextion sensitivity may not necessarily be accompanied by a loss in quality discrimination for suprathreshold odors.

As is true for other senses, olfactory abilities rely on both sensory and cognitive processes. Detextion and quality discrimination are often referred to as tasks that mainly draw on sensory abilities, and perceived odor intensity, while driven by the magnitude of the stimulus, also provides a reflection of sensory acuity. Odor identification requires some detextion sensitivity (assumed to underlie relatively strong odor sensations), quality discrimination, and cognitive or transmodal abilities such as recognition memory, odor-visual integration, semantic knowledge, or word retrieval (Hedner et al., 2010; Larsson et al., 2005; Olofsson & Gottfried, 2015), which all are important for human daily routines (Croy et al., 2014). As would be expected based on their declining olfactory abilities, aging persons are often clearly impaired in odor identification tasks (Hedner et al., 2010; Hummel et al., 1997; Kern et al., 2014; Larsson et al., 2000, 2004; Makowska et al., 2011; Menon et al., 2013; Olofsson et al., 2010; Pinto et al., 2014; Schubert et al., 2011; Wehling et al., 2011; Wilson et al., 2011; Wong et al., 2010). Prevalence rates for olfactory dysfunction, based on cued odor identification, increase from 11% to 24% in middle-aged individuals to 37% to 70% at the age of 70 years (Brämerson et al., 2004; Murphy et al., 2002; see also Choi et al., 2018; Hoffman et al., 2016; Seubert et al., 2017). Results from longitudinal studies suggest that these findings cannot simply be attributed to cohort effects (Schubert et al., 2011; Wehling, Lundervold, et al., 2016). Other cognition-dependent olfactory functions impaired in aging are episodic odor recognition memory, perception of odor familiarity, and edibility judgments (Larsson & Bäckman, 1993, 1997; Murphy et al., 1997; Wehling, Wollschlaeger, et al., 2016). Olfaction thus involves many facets, most of which are challenged by aging processes.

In accordance with results on orthonasal olfaction, aging-related impairments in retronasal olfaction are observed in detextion, quality and intensity discrimination (Cain et al., 1990; Duffy et al., 1999; S. Koskinen & Tuorila, 2005; J. C. Stevens & Cain, 1993), perceived intensity (J. C. Stevens & Cain, 1986), and identification (Murphy, 1985). Furthermore, de Graaf et al. (1994) reported that aging adults, on average, perceive high concentrations of food flavors to be less intense than do younger adults, and that the optimal pleasantness concentrations of the flavors are higher in old age.

Persons with declining olfactory functions often report a diminished quality of life, negative effects on interpersonal relations, worries about not perceiving toxic substances, poor control over personal hygiene, difficulties with daily routines, and depression (Boesveldt, Postma et al., 2017; Croy et al., 2014). It is unfortunately common among aging persons to be unaware of having poor olfactory abilities (Bahar-Fuchs et al., 2011; Nordin et al., 1995; Rawal et al., 2014; Seubert et al., 2017; Wehling et al., 2011, 2015; White & Kurtz, 2003). Recent longitudinal research, however, provides a slightly more optimistic view, as self-reported olfactory decline often reflect objective olfactory decline, at least in cognitively intact aging adults (Ekström et al., 2019). Nevertheless, a rather large proportion of aging adults may constitute a risk group with respect to safety issues (e.g., detextion of smoke, gas leaks, and spoiled food).

The age-related changes in olfactory function described earlier depend on anatomical and physiological changes such as a decreased number of olfactory receptor cells, reduced volume of olfactory brain regions such as the olfactory bulb, amygdala, piriform cortex, anterior olfactory nucleus, and frontal poles as well as broader electrophysiological tuning curves for receptor cells (Attems et al., 2015; Doty & Kamath, 2014; Rawson, 2006). Aging-related olfactory loss is also associated with cortical processing changes, such as a diminished cortical processing speed (Lötsch & Hummel, 2006; Murphy et al., 2000; Morgan & Murphy, 2010; Stuck et al., 2006) and decreased odor-elicited activity in the enthorinal cortex, piriform cortex, amygdala and periamygloid cortex, hippocampus and parahippocampal gyrus, orbitofrontal cortex, and insula and perisylvian zones (Cerf-Ducastel & Murphy, 2003; Suzuki et al., 2001; Yousem et al., 1998; Wang et al., 2005, 2017). Regarding age-related neurochemical changes, acetylcholine is of particular interest as it is a neurotransmitter critical for the olfactory system and has shown deficiency in the aging brain (Doty & Kamath, 2014). Furthermore, nigrostriatal dopaminergic degeneration (Wong et al., 2010) and age-related reductions in the binding potential for the striatal dopamine transporter in putamen (Larsson, Farde, et al., 2009) may be linked to olfactory decline in aging.

Despite the multifaceted nature of age-related olfactory loss, it may be at least partly avoidable; so-called successfully-aged persons have a reduced risk of age-related impairment in olfactory detextion sensitivity (Nordin et al., 2012). In line with this finding, poorer odor sensitivity has been reported in institutionalized compared with independently living aging persons (Sulmont-Rosse et al., 2015). Based on what is known about olfaction and aging, we will review five domains where the age-associated olfactory dysfunction is of particular interest, and where more research is needed to facilitate olfactory-related health and well-being in the aging population.

## Nutrition and Health

Malnutrition is a major concern regarding the aging population, in particular among the institutionalized aging persons. Between 3% and 10% of independently living aging adults and 25% to 60% of institutionalized aging adults suffer from malnutrition. The problem is particularly common in nursing homes, with prevalence rates of up to 85% (Saletti et al., 2000; Swedish National Food Administration, 1998; Vellas et al., 2001). Malnutrition among aging adults can largely be attributed to aging-related anorexia, which is due to various social, psychological, and biological factors that include disease (Brownie, 2006; Donini et al., 2003).

The olfactory system plays key roles in determining when we decide to start eating, how much to eat, and what to eat. The onset is partly determined by hunger and food cravings. Food odors encourage food intake, for example, by secreting insulin that lowers the blood-glucose level and generates a hunger sensation. The odors may also trigger secretion of saliva, gastric acids, and enzymes as well as cardiovascular and thermal responses to prepare for metabolization (Richardson et al., 1977). So-called learned sensory control has been associated with onset of a meal in which food cravings are evoked by classical conditioning (De Houwer et al., 2001). During a meal, some mechanisms enhance continued eating, whereas others enhance its termination (Davis & Levine, 1977). The former group includes odor sensations that release dopamine, activating the “reward system,” and serotonin, evoking well-being to encourage continued eating (E. T. Rolls, 2015). The orbitofrontal cortex, insula, and amygdala play important roles in this context. Learned sensory control can contribute to termination of the meal. For example, it was early shown that calorie content can be predicted based on the flavor, and that the meal size can be adjusted depending on the flavor (Birch & Deysher, 1986). Finally, food preference and aversions strongly affect what we decide to eat, explained by classical and instrumental conditioning. We learn early in life, primarily based on olfactory cues, to identify an item with respect to its edibility (Schaal et al., 1998). Preferences begin to form even before birth by flavors in the amniotic fluid from the mother’s food intake (Mennella & Beauchamp, 1993; Mennella et al., 1995). Regarding food aversions, olfactory signals evoke nausea and vomiting that underlie the aversions, and odor-based associations of this kind appear to be the most common reason for a food aversion (Nordin et al., 2004).

Age-related changes in sensory perception are likely to contribute considerably to anorexia of aging (MacIntosh et al., 2000). Perception of food is multisensory, involving olfaction, gustation, chemical and nonchemical skin senses, vision, audition, and kinesthesis; these senses provide information about the food’s flavor, temperature, color, appearance, and texture. Olfaction appears to shape food choice and intake, whereas taste has an important role as a macro-nutrient sensing system (Boesveldt & de Graaf, 2017).

Given the importance of olfaction for eating behavior, its impairment may be accompanied by impact on food intake and health. This is indeed the case, with poor food appreciation and decreased appetite resulting in change in body weight (Croy et al., 2014). Aging individuals, and in particular the institutionalized, are considered especially vulnerable in this respect. Thus, researchers recognized decades ago that changes in chemosensory perception in aging are associated with poor food appreciation and appetite, change in food choice such as decreased dietary variation, poor nutritional status, change in body weight, and increased risk for chronic disease (Brown, 1976; Duffy & Bartoshuk, 1996; Ferris & Duffy, 1989; Griep et al., 1995; Mattes-Kulig & Henkin, 1985; Schiffman & Zervakis, 2002; D. A. Stevens & Lawless, 1981; Wysocki & Pelchat, 1993; however, see also Arganini & Sinesio, 2015). A more recent study, using longitudinal data, strengthens the associations by showing that olfactory impairment in aging persons, at least among women, might be a contributing factor to poor diet quality (Gopinath et al., 2016). Furthermore, B. J. Rolls and McDermott (1991) demonstrated diminished sensory-specific satiety in old age, which may contribute to the decreased dietary variation with age, and Kremer et al. (2014) showed that those with diminished olfaction more often report eating the same meal within a week. However, not all studies have shown an association between chemosensory impairment and nutritional problems (e.g., Toussaint et al., 2015), and lack of sensory feedback from eating may in some cases actually lead the individual to eat more, increasing risk for obesity. There is also a considerable risk among aging adults to ingest spoiled food. It has, for example, been suggested that aging adults are less likely than young adults to reject foods with unpleasant odors (Pelchat, 2000). The unawareness of age-related olfactory loss (e.g., Nordin et al., 1995; Seubert et al., 2017) may aggravate the risk of ingesting spoiled food as these persons are less likely to take precautions to avoid eating such food.

How should these risks be mitigated? Early work by Schiffman et al. demonstrated that anorexia in the aging population may remit when foods are amplified by additional flavoring to compensate for diminished chemosensory function, resulting in increased preference for and intake of food, increased salivation, and improved immunological status and grip strength (Schiffman, 1998; Schiffman & Miletic, 1999; Schiffman & Warwick, 1988, 1993). However, in a recent review, Boesveldt et al. (2018) conclude that studies on flavor enhancement provide mixed results regarding its positive impact on food preference and intake among the aging adults. Because of the personal nature of food preferences and the heterogeneity of chemosensory decline in the aging population, flavor enhancement may not fit an individual’s expectations or preferences (S. Koskinen et al., 2003). Future research on this topic should consider such individual differences; declining sensory abilities may interact with several other psychological and health-related variables that may moderate the risk for malnutrition and other harmful food behaviors. As the olfactory epithelium microbiome composition may be important for olfactory function and BMI, future work should assess whether it might play a causal role in whether olfactory abilities decline or are preserved in the aging process (K. Koskinen et al., 2018).

## Cognition and Dementia

Olfactory deficits are a common feature of the normal aging processes but may also reflect unique patterns of cognitive brain changes in aging. To date, several studies have shown that olfactory deficits often coincide with or even precede impairments in nonolfactory cognitive tests (Djordjevic et al., 2008; Dulay & Murphy, 2002; Swan & Camelli, 2002; Wilson et al., 2006). Correlations between olfactory and cognitive deficits in aging adults are unlikely to arise simply due to the semantic memory components in the olfactory identification task. In fact, word knowledge is typically retained in old age, whereas odor identification declines (Larsson, Hedner, et al., 2009). Evidence from a large (*n* = 2,280) population-based sample of participants aged 60 to 100 years suggested that memory-based olfactory tests (odor recognition memory and identification) were differentiated from verbal episodic and semantic memory domains, and odor memory was best conceptualized as a distinct and separate factor in old age (Larsson et al., 2016). This outcome suggests that higher order olfactory functions have unique features relative to other types of declarative memory in old age. Indeed, the results favor the notion of a sensory system driven by its own premises, as exemplified by a poor language integration (Olofsson et al., 2014), low imagery capacity (Arshamian & Larsson, 2014), and a higher emotionality relative other sensory modalities (Arshamian et al., 2013).

 Prospective studies show that performance in odor identification could predict future decline in general cognition (Conti et al., 2013; Graves et al., 1999) or executive functioning (Schubert et al., 2013), and effects persist even after controlling for vocabulary, a nonolfactory control task where synonyms are matched (Olofsson, Larsson et al., 2020; Olofsson et al., 2009). These previous findings have recently been confirmed by a large-scale population-based study of middle-aged and aging participants (Tebrügge et al., 2018). Here, participants with olfactory deficits performed significantly worse on a large variety of cognitive subtests, such as verbal memory and fluency, problem solving, visuo-spatial abilities, speed of processing, and inhibition. According to some studies, the risk of future cognitive decline in aging individuals with olfactory loss depends on the ApoE gene; a plasma protein involved in lipid transport, and located on chromosome 19, carrying the three possible alleles ε2, ε3, and ε4. The gene is expressed in the central nervous system (CNS), including the olfactory bulb and the olfactory epithelium (Nathan et al., 2007; Struble et al., 1999), and is believed to play a role in neuronal regenerative processes as well as in the development of Alzheimer’s disease (AD). Aging ApoE ε4 carriers with no sign of dementia have a diminished olfactory ability relative to noncarriers (Graves et al., 1999; Larsson et al., 2016; Olofsson et al., 2010), and in the ε4 carrier group, olfactory deficits are more strongly indicative of generalized cognitive decline in the coming years (Olofsson, Larsson et al., 2020; Olofsson et al., 2009). Aging adults with an ε4 allele also show a diminished electrophysiological brain activity in response to odors (Green et al., 2013). Despite some inconsistency in the literature (Handley et al., 2006), impaired odor identification has been observed in cognitively impaired aging ε4-carriers without dementia (Murphy et al., 1998). Longitudinal data suggest that aging ε4-carriers show larger-than-normal decline in odor identification performance (Calhoun-Haney & Murphy, 2005; Josefsson et al., 2017). The accelerated decline concurs with declining episodic memory abilities (Josefsson et al., 2017). The main interpretation of these findings is that in the ε4 carriers, olfactory dysfunction is predominantly caused by early, still preclinical AD pathology which accumulates in the mediotemporal lobe (Olofsson et al., 2016).

It was early demonstrated that pathology in AD encompasses olfactory brain areas as well as adjacent regions in the medial temporal lobes (e.g., Braak & Braak, 1997). Moreover, poor performance on standardized olfactory tests correlates with dementia-related pathological markers in the brain of AD patients, such as with *t*-tau concentrations in cerebrospinal fluid (Reijs et al., 2017). Olfactory abilities are often correlated with those cognitive functions that typically decline in AD. Several prospective studies have suggested that olfactory loss might be particularly associated with future dysfunctions in tasks assessing episodic memory, which are early symptoms of AD (Finkel et al., 2011; Roberts et al., 2016; Swan & Carmelli, 2002; Wilson et al., 2006). Accordingly, there is strong evidence for olfaction being markedly impaired in patients with AD (Devanand, 2018; Sun et al., 2012). A meta-analysis, including over 50 studies, showed that olfactory deficits were present in AD patients relative to controls in all included studies (Rahayel et al., 2012). Deficits in cognitively demanding olfactory tasks, such as identification and recognition memory, had higher effect sizes than deficits in olfactory detextion sensitivity.

It has been proposed that olfactory deficits may be indicative of later conversion to AD. Although the exact cause of the disease remains elusive to this day, AD pathology such as *t*-tau and amyloid plaques are associated with neuronal loss and may affect the brain many years before a diagnosis can be given. The correct identification of individuals who are within the preclinical phase of dementia will be of key importance once successful methods to slow down disease progression are established. In recent years, a growing number of prospective research studies have focused on whether olfactory deficits may be predictive of later conversion to AD. Mild cognitive impairment (MCI) is a syndrome characterized by cognitive decline greater than can be expected based on a person’s age and educational background, constituting a stage between normal cognitive aging and dementia (Gauthier et al., 2006). While the progression rate to AD is notably higher among patient groups with MCI, as compared with cognitively intact aging persons, not every person that has been diagnosed with MCI will eventually convert to dementia (Gauthier et al., 2006). MCI patients often perform poorly on olfactory tests (e.g., Goette et al., 2018; Palta et al., 2018), which raises the question if olfactory assessments can be used to predict whether an MCI patient will eventually develop dementia. In recent years, a handful of prospective longitudinal studies have therefore attempted to investigate the predictive role of olfactory testing for AD. All studies found significant associations between baseline odor identification ability in MCI and risk of later dementia progression (Conti et al., 2013; Devanand et al., 2008, 2015; Roberts et al., 2016). Devanand et al. (2008) estimated odor identification testing to have a sensitivity rate of almost 50% for predicting AD conversion within 3 years.

Apart from studying MCI patient groups, a few population-based studies have so far investigated the predictive utility of olfactory baseline ability on conversion to AD (Adams et al., 2018, Devanand et al., 2015; Stanciu et al., 2014; Yaffe et al., 2017). In these studies, a total of 7620 participants were followed longitudinally over an average follow-up time span of 94 months. The studies consistently found that poor olfactory identification predicted later progression to AD. All studies included follow-up analyses that controlled for the potentially confounding effects of demographic variables and baseline cognition, but the association between olfactory function and AD persisted. Stanciu et al. (2014) were the first to report a significant association between baseline odor identification performance and conversion to AD within 10 years in community-dwelling aging adults. Similar results were obtained by recent studies based on U.S. samples; effects are present in subsamples of Black and White persons, but the predictive effects are slightly weaker in the Black subgroup (Yaffe et al., 2017). Overall, poor odor identification is associated with conversion to AD within 2 to 10 years, with effect sizes that compare well to the standard AD biomarkers in MCI patients (Adams et al., 2018; Devanand et al., 2015). Although these group-level results are promising, it is important to note that such predictions are far from certain on the individual level. Most likely, this is because many aging persons have an olfactory impairment for reasons that are unrelated to dementia and which were described earlier. Thus, Devanand et al. (2020) recently suggested, based on empirical results from a large-scale longitudinal study, that olfaction should be used as a screening tool, such that aging persons with a high olfactory performance can be regarded as being at very low risk for dementia in the coming years, whereas a poor olfactory performance is less informative in terms of dementia risk (because olfactory impairment has many other potential causes), and further investigation is always needed before an increased risk of dementia can be established.

Although this section has focused on MCI and AD, where olfactory impairments are closely linked to cognitive decline, olfactory loss is also prevalent in several other neurodegenerative disorders and dementia syndromes. After AD, vascular dementia (VaD) is a common cause. Nevertheless, studies about olfactory deficits in VaD are still relatively sparse. Although olfaction appears to be impaired in VaD, mixed findings have been obtained regarding the magnitudes of these effects (Duff et al., 2002; Gray et al., 2001; Knupfer & Spiegel, 1986). An explanation for this discrepancy has been proposed by Alves et al. (2014), who suggest that the extremity of an olfactory deficit may depend on the location and extent of vascular effects in the brain, which is highly variable among VaD patients. By contrast, olfactory dysfunction is among the earliest nonmotor symptom of Parkinsons’ disease (PD) and is prevalent in approximately 90% of early stage PD cases (Doty, 2012). A meta-analysis comparing the effect of AD and PD on olfactory functions showed that both patient groups were more impaired in identification and recognition tasks than odor threshold tasks. However, PD showed larger impairments than AD patients in the detextion task. This outcome suggests that persons with PD may be more impaired in sensory-driven tasks, whereas AD patients show larger impairments in higher order olfactory functions (Rahayel et al., 2012). In recent work, Lee et al. (2020) compared resting-state olfactory functional networks in PD and AD. Interestingly, comparisons showed that PD exhibited lower olfactory bulb connectivity with striatal-thalamic-frontal regions and lower orbitofrontal connectivity with striatal-frontal regions than AD. Overall, these pattern of findings are indicative of different neural mechanisms for smell dysfunction in PD and AD. In fronto-temporal dementia, a clinical spectrum that includes a variety of disorders, olfactory-cognitive (but not sensory) deficits are often observed alongside severe nonolfactory symptoms (Silva et al., 2019). The type of olfactory impairment depends largely on the location of cortical atrophy (Olofsson et al., 2013). The most well-established findings are the odor identification and naming deficits that co-occur with other semantic or associative deficits in patients with frontal or temporal lobe atrophy (Olofsson et al., 2013; Silva et al., 2019).

In summary, the results from recent prospective studies indicate that olfactory deficits may represent a promising candidate marker for preclinical AD, both in patient groups with MCI and in the general population of aging adults without an established cognitive impairment at baseline. Future studies are needed to investigate how early identification of AD may benefit from including olfactory assessments alongside other possible and established biomarkers. Of particular interest is to develop methods to rule out olfactory impairments due to other causes (e.g., postviral olfactory loss) in order to enhance the predictive utility of olfactory assessments.

## Mortality

In the last decade, prospective studies indicate that olfactory deficits may be related to an elevated risk of mortality in aging persons. Wilson et al. (2011) found that the risk of death within an average time period of 4 years decreased by about 6% for each additional correct choice on an odor identification task. Similarly, Pinto et al. (2014) reported that anosmic individuals were more than 3 times as likely to die within 5 years as compared with normosic participants, even after statistically adjusting for numerous demographic, cognitive, and health variables. Gopinath et al. (2012) reported a similar association between olfactory deficits and risk of death. However, the results of their study also suggested the effect might be driven by participants who already had a cognitive impairment at baseline. An important emerging question has thus been whether the association between olfactory performance level and later mortality risk may be due to dementia conversion after baseline and before death at follow-up. While the three aforementioned studies were not able to account for dementia conversion, two recent large-scale population-based studies were able to analyze whether or not the association between olfactory deficits and mortality risk was driven by aging persons who converted to dementia after baseline and before death at follow-up. Devanand et al. (2015) followed 1,169 community-dwelling aging persons for a time span of approximately 5 years and found olfactory dysfunction to be predictive of a higher mortality risk even after statistically controlling for dementia conversion after baseline. Similar results were obtained by Ekström et al. (2017) in a sample of 1,774 middle-aged and aging adults in Sweden. The authors found olfactory deficits to emerge as a predictor of mortality risk during a time span of nearly 10 years, independently of dementia conversion between baseline and follow-up. Furthermore, the authors reported that the association between olfactory deficits and risk of mortality was present also in middle-aged adults who were younger than the participants in previous studies. These results recently received further empirical support in large-scale studies (Choi et al., 2020; Liu et al., 2019).

Taken together, these studies suggest that olfactory deficits may be related to an increased risk of mortality in both middle age and in aging. Of particular interest for future research is the mixed findings regarding dementia conversion, which does not account for the association between olfactory deficit and mortality in Ekström et al. (2017), but plays a role in some of the deaths in the research by Liu et al. (2019). Thus, the question of what exact mechanism(s) underlie the mortality–olfactory relationship remains unresolved. In some individuals, an olfactory deficit may pose a lethal hazard, as it may increase the risk of accidents at home, such as gas poisonings and the consumption of spoiled foods (Bonfils et al., 2008; Doty & Kamath, 2014; Santos et al., 2004). However, it is unlikely that olfactory-related accidents, a relatively rare cause of death among aging persons, may emerge as a main explanation. Furthermore, malnutrition caused by olfactory loss may lead to increased risk of death, although this explanation has not received empirical support thus far (Pinto et al., 2014).

 An alternative explanation is that the olfactory system may be particularly exposed to the effects of aging and disease as it relies on cellular regeneration (Pagano et al., 2000), a process that has been associated with age-related impairment (Laws et al., 2002; Striepens et al., 2011; Watabe-Rudolph et al., 2011). Furthermore, the olfactory system is unique among sensory systems in being directly exposed to the environment. As such, it may be sensitive to accumulative damage caused by environmental hazards (Doty, 2008). Thus, rather than causing death per se, olfactory performance may be indicative of fundamental physiological processes of aging in the CNS and their interactions with environmental exposures (Pinto et al., 2014). As a consequence, the state of the olfactory system may be sensitive to a variety of health issues that might increase mortality risk. More research is needed to establish these mechanisms in more detail (Van Regemorter et al., 2020).

## Environment and Genetics

Fundamental to our understanding of age-related olfactory changes is the interplay between environment and genetics. Research on the heritability of olfactory functions suggest that olfactory abilities do not have a strong heritability component, and that most variation can be attributed to the environment (Doty et al., 2011; Finkel et al., 2001). Although odor identification has a modest heritability, due to its partial reliance on overall cognition (Finkel et al., 2001), aging individuals show a low heritability, suggesting environmental effects are paramount. Among the environmental effects on olfaction in old age, medical conditions and medication are among the most important. Doty (2008) listed over 200 etiologies that, irrespective of age, can cause an olfactory disorder. The mechanisms underlying these disorders can be classified as conductive (e.g., caused by nasal polyposis), sensorineural (e.g., postviral upper respiratory infection), or impairment in the olfactory CNS (e.g., tumor). The most common etiologies of olfactory disorders among patients seeking medical attention at ear, nose, and throat clinics include upper respiratory infection, chronic sinusitis, nasal polyposis, allergic rhinitis, and head trauma (Brämerson et al., 2007; Temmel et al., 2002). The presence of type-2 diabetes accelerates age-related olfactory decline (Ekström et al., 2020; Olofsson et al., 2010). Given the profound impact of such conditions on the olfactory system, it is not surprising that environmental variables overshadow genetic variables in their effects of olfactory abilities among aging individuals (Doty & Kamath, 2014; Rawson, 2006).

Medication is an environmentally related factor of particular relevance in the context of olfaction and aging. Results from the United States suggests that adults over 65 years of age take on average about three to four medications and institutionalized aging persons take about twice as many (Finkelstein & Schiffman, 1999). It has been suggested that more than 250 drugs may alter smell and taste sensations. These include antihistamines, lipid-lowering drugs, antimicrobial medications, antineoplastic medications, asthma medications, antihypertensives, muscle relaxants, and antidepressants (Schiffman, 1991). Nutritional deficits, such as reduced levels of zinc and vitamin A and B, are known to affect sensitivity and hedonics of taste and smell (Aliani et al., 2013; Schiffman, 1997). The effects of malnutrition on chemosensory perception may, in turn, affect food intake and aggravate the state of malnutrition. Poor oral health in aging persons, such as tooth loss and caries, may affect retronasal olfactory perception and provides an additional challenge (Seiberling & Conley, 2004; Ship, 1999; Solemdal et al., 2012).

Long-term exposure to environmental pollutants, such as air pollution, may constitute a key environmental variable in age-related olfactory decline. Microscopic airborne pollutants can bypass the blood–brain barrier via the olfactory nerves, where they may initiate inflammation (Block & Calderón-Garcidueñas, 2009; Lucchini et al., 2012; Oberdörster et al., 2004). It has therefore been proposed that exposure to airborne pollutants may affect olfactory function in aging adults for whom xenobiotic exposure has accumulated over a longer time (Doty & Kamath, 2014). Indeed, one study has so far found significant associations between exposure to the fine particulate matter (PM_2.5_, diameter <2.5 μm) and worse olfactory identification scores among community-dwelling aging adults (Ajmani, Suh, Wroblewski, et al., 2016). These findings are corroborated by studies based on proxies for pollutant exposures (Ajmani, Suh, & Pinto, et al., 2016; Calderón-Garcidueñas et al., 2010; Hudson et al., 2006; Ranft et al., 2009).

In the last few years, researchers have begun to investigate olfactory abilities using genome-wide associations. Human olfactory receptor genes are characterized by high levels of genetic inactivation and variability, which might lead to highly variable odor percepts (Mainland et al., 2014). Indeed, Gisladottir et al. (2020) recently found that the intensity and nameability of individual food-associated odors such as trimethylamine (fish odor), trans-anethole (liqorice odor), and trans-cinnamaldehyde (cinnamon odor) might vary in the population due to genetic influences on olfactory receptors. Although these results highlight some ways by which human genetic variability might shape olfactory abilities, their implications for aging are less well known. There is still little work done on gene–environment interactions from an aging perspective, for example, how genetics might make certain aging individuals more vulnerable to olfactory deficits as a function of medication, less able to recover from post-viral anosmia, or other common causes of age-related olfactory loss. Such approaches would, however, be beneficial for our basic understanding of the aging olfactory system, and they might have clinical usefulness.

## Training-Based Enhancement

The olfactory decline observed in aging may seem irreversible. But emerging evidence suggests that olfactory performance may in fact be trained. Most olfactory training studies to date have focused on patients with severe olfactory loss following a viral infection or other causes, and the studies have involved a simple training protocol where a set of odors are smelled twice daily (Hummel et al., 2009). Participants in these studies are usually of middle age or aging, and many appear to benefit from the olfactory training regime relative to a control group that might experience spontaneous recovery but no training effect (Damm et al., 2014). One study suggested that the benefits of olfactory training extend to nonolfactory health-associated variables; after olfactory training, participants reported feeling younger than before training (Wegener et al., 2017). The study also reported cognitive performance benefits of olfactory training, but this interaction effect was driven by poorer baseline performance of the olfactory training group so it needs replication before conclusions can be drawn about cognitive effects. Indeed, if olfactory training leads to cognitive benefits, what are the mechanisms? While only a few research reports have addressed cortical changes related to olfactory training, they have yielded promising results in young adults. A study on cortical thickness suggested that olfactory training led to increased thickness not only in key olfactory structures but also in fronto-temporal areas outside of the olfactory cortex (Al Aïn et al., 2019). A similar study on odor identification training showed functional brain activity changes in a fronto-parietal network associated with higher cognitive abilities (Fournel et al., 2017). Connectivity analysis might provide a further understanding of how dynamic cortical networks are modulated by repeated olfactory task engagement and how they might underlie behavioral changes (Reichert & Schöpf, 2018). The results so far are promising, but further research should focus on achieving high-powered studies with aging participants, who might be less susceptible than young adults to training effects.

The notion that olfactory training has potential to not only help recovery from age-related olfactory loss but also boost cognitive and emotional health of aging participants is exciting. However, to substantiate claims of cognitive and health-related effects, we argue that researchers should adopt more sophisticated training paradigms and pinpoint the biological mechanisms that may mediate generalizable benefits of olfactory training. Well-calibrated nonolfactory control conditions and rigorous assessment of transfer properties from olfactory training to nonolfactory cognitive benefits are often lacking. While new methods are emerging, as discussed earlier, the standard olfactory training methodology involves merely a focused smelling and does not try to engage olfactory–cognitive processes. The olfactory exposure is thus relatively passive in comparison to typical visual cognitive “brain training” training paradigms. Furthermore, the standard olfactory task is not behaviorally well-characterized as no precise behavioral data are collected during the training period, which might last for over 6 months. Little is known about how participants engage with the odors during training, and this limits our understanding of the training effect. For this reason, we have developed new methods for olfactory training that may facilitate research progress in this area. We recently developed a smell memory game that involves finding 12 matching pairs among a set of 24 stimuli in as few trials as possible (Olofsson et al., 2020). Healthy adult participants were randomized to train either their smell memory using tea flavors, or visual memory using Asian language symbols, for 40 days. Results showed that olfactory training, but not visual training, leads to improvements in odor discrimination and naming, as assessed with the Sniffin sticks olfactory assessment. The performance increase put the participants at the same level as a group of wine experts. Furthermore, we found that olfactory training led to performance increase in the visual task (i.e., cross-sensory transfer), whereas no corresponding benefit was observed in the olfactory task from visual memory training (Olofsson, Ekström et al., 2020). This task allows for assessing and logging performance at each training occasion. Game-like tasks might become a useful mode of training, especially for aging individuals who have retained some olfactory sensitivity, but who wish to use olfactory training to improve further, or to yield possible cognitive or emotional benefits. Ongoing efforts include assessing how well the smell memory training paradigm works in aging participants and how training may lead to performance and cortical changes in this group, and computerized versions of this task that enable laboratory testing of how cortical processing changes mediate performance changes. A further development which might accelerate olfactory training research is innovative hardware and software solutions for computerized olfactory training. Computerized odor interactions may allow for more precise behavioral assessment, game-like interfaces with adaptive difficulty level and feedback that increases motivation. These features can be used to facilitate user engagement and rehabilitation. Toward this goal, we are developing hand-held olfactometers for Virtual Reality applications (Niedenthal et al., 2019), as well as simpler olfactory hardware solutions that can be used to run olfactory training and assessment on a laptop computer (Niedenthal et al., 2021). Olfactory deficit is a common consequence of coronavirus infection and has recently received increasing attention. As aging adults are dysproportionately affected by the virus and olfactory abilities are already fragile in many aging persons, the coronavirus might have long-lasting and detrimental effects on olfaction in the aging population. Improving olfactory training methodology is challenging because of the compound difficulties of studying olfaction remotely, and of randomized controlled trials, but these challenges need to be met in order for the field to move forward (Pekala et al., 2016).

## Conclusion

Much progress has been made in understanding olfactory impairments in aging. Although these impairments arise from a multitude of factors, they are not unavoidable. We argue that future research challenges appear at the intersection of biology and behavior and should aim at reversing olfactory impairments and its health-associated outcomes in aging individuals whenever possible. Health-related consequences of olfactory loss are heterogeneous, and little is still known about individual differences that determine outcomes such as malnutrition. Olfactory loss is a well-established risk factor for dementia and cognitive decline in aging individuals. Here, optimizing the predictive utility of olfactory tests for dementia, with a special focus on how to rule out more benign causes of olfactory loss, remains an issue of critical importance. Olfactory loss has also emerged as a predictor of mortality risk, but little is still known about the underlying mechanisms, and to what extent dementia is a mediating factor that explains the association between olfactory loss and mortality. Given the wide-ranging health aspects of olfactory loss, it is critical to develop effective treatments for olfactory loss. Smell training offers a set of noninvasive methods by which olfactory exposure may benefit olfaction and perhaps also health and cognition. Future research may explore the many possible benefits of smell training; however, this line of research will benefit from improved methodologies using computer-based assessments and other innovations for improving transparency, compliance, effectiveness, and precision. Research on olfactory genetics has led to an appreciation for the diversity and individual differences in olfactory receptor biology, but we still have a limited understanding of how these genetic variables may have different effects in aging individuals and how genetic effects shape our resilience to environmental effects that accumulate over the lifespan. In sum, understanding how olfactory abilities are diminished and how they may perhaps be alleviated or recovered in old age presents a set of challenging tasks for researchers in the years to come.
